# Supplementary material for: Association between oral microbiome and seven types of cancers in East Asian population: a two-sample Mendelian randomization analysis
Source: Front Mol Biosci. 2023 Nov 21;10:1327893. doi: 10.3389/fmolb.2023.1327893 (PMC10702768; doi:10.3389/fmolb.2023.1327893)
Supplement: Supplementary file 1 [file DataSheet1.ZIP › Supplementary Materials/MR plots for tongue/tongue═╝/Colorectal cancer/pheno.2764_to_colorectal cancer_leave_one_out.pdf]

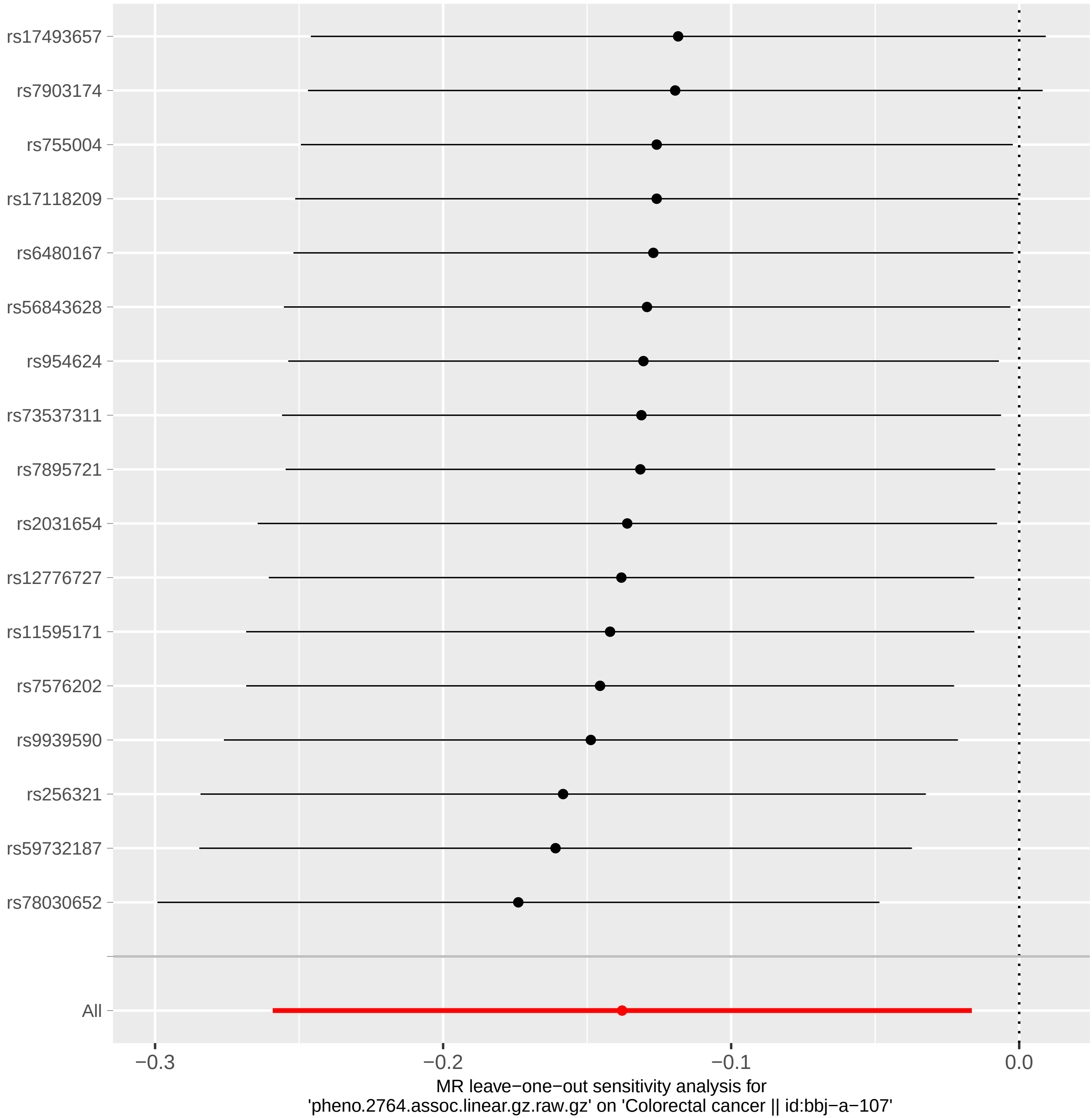
rs17493657
rs7903174
rs755004
rs17118209
rs6480167
rs56843628
rs954624
rs73537311
rs7895721
rs2031654
rs12776727
rs11595171
rs7576202
rs9939590
rs256321
rs59732187
rs78030652
All
−0.3
−0.2
−0.1
0.0
MR leave−one−out sensitivity analysis for
'pheno.2764.assoc.linear.gz.raw.gz' on 'Colorectal cancer || id:bbj−a−107'
